# Sex differences and gender-oriented characteristics in intensive care unit admissions for patients with traumatic brain injury in Spain

**DOI:** 10.3389/fmed.2025.1622422

**Published:** 2025-09-26

**Authors:** Manuel Cruz-Garcinuño, Raúl Juárez-Vela, Elena Chover-Sierra, Noelia Navas-Echazarreta, Michal Czapla, María Luisa Ballestar-Tarín, Raquel-María Martínez-Pascual, Kapil Nanwani-Nanwani, Ainhoa Serrano-Lazaro, Ismael del Val-Rey, Manuel Quintana-Díaz, Antonio Martinez-Sabater

**Affiliations:** ^1^Doctoral Program in Medicine and Surgery, Autonomous University of Madrid, Madrid, Spain; ^2^Faculty of Health Sciences, Research Group in Care, University of La Rioja, Logroño, Spain; ^3^Idi Paz Research Institute, PBM Group, Madrid, Spain; ^4^Faculty of Nursing and Podology, Nursing Care ad Education Research Group (GRIECE), University of Valencia, Valencia, Spain; ^5^Department of Internal Medicine, Consorcio Hospital General Universitario de Valencia, Valencia, Spain; ^6^Department of Emergency Medical Service, Faculty of Nursing and Midwifery, Wroclaw Medical University, Wroclaw, Poland; ^7^Intensive Care Unit, Hospital La Paz, Madrid, Spain; ^8^Intensive Care Unit, Hospital Clínico Universitario de Valencia, Valencia, Spain; ^9^Care Research Group (INCLIVA), Hospital Clínico Universitario de Valencia, Valencia, Spain

**Keywords:** critical care, epidemiology, craniocerebral trauma, trauma, brain injuries

## Abstract

**Background:**

Traumatic brain injury (TBI) is a major public health concern with significant mortality, disability, and socioeconomic impact. Previous studies have shown that biological sex influences TBI incidence and outcomes, yet sex-specific data remain underexplored. We aimed to analyze clinical characteristics, resource utilization, and outcomes of ICU-admitted TBI patients in Spain, with a focus on sex-related differences.

**Material and methods:**

We performed an observational and retrospective study utilizing data from the RETRAUCI registry, involving 50 registered ICUs and 124 investigators. Patients admitted from March 2015 to December 2019 with isolated significant TBI (AIS ≥ 3) were included. Data on epidemiology, acute management, injury type, resource utilization, complications, and outcomes were recorded.

**Results:**

Of the 950 patients, 76% were male. Women had significantly longer ICU stays (11.03 vs. 9.43 days; *p* = 0.026), and a higher rate of chronic psychotropic drug use. Men were more frequently involved in traffic accidents and had higher rates of alcohol (23.2% vs. 9.3%; *p* < 0.001) and drug use (11.4% vs. 5.3%; *p* = 0.007). No significant sex differences were found in complication rates, neuromonitoring, or mortality.

**Conclusion:**

These findings show that sex significantly influences TBI patterns and in-hospital evolution. Men had higher rates of trauma from risk-related behaviors, while women experienced longer ICU stays and greater psychotropic use. Such differences call for sex-specific approaches in clinical care and further prospective research.

## 1 Introduction

Traumatic brain injury (TBI) is defined as an alteration of brain anatomy and function caused by violent exchanges of mechanical energy (1). It is one of the leading causes of morbidity and mortality worldwide, accounting for approximately one-third of injury-related deaths in the United States (2). In Spain, the annual incidence of TBI is estimated at 200 new cases per 100,000 inhabitants (3–5). From an epidemiological perspective, TBI is one of the main causes of trauma-related death globally, with an overall rate of 579 per 100,000 people per year. Its main causes include falls and traffic accidents, with incidence varying according to factors such as sex, age, and socioeconomic level, being more prevalent in developing countries (6–8). Falls are the leading cause of TBI, followed by motor vehicle accidents (9).

Traumatic brain injury (TBI) is a critical public health issue, with significant impacts on mortality and disability worldwide. Its high prevalence and socio-familial repercussions have raised concerns within health systems (10). By 2030, it is estimated to surpass other pathologies as a cause of death and disability, posing a major challenge in healthcare costs and the adaptation of therapeutic strategies and the development of new diagnostic tools (2). In countries like Canada, the economic costs of TBI are projected to exceed 8 billion dollars by 2031 (11–15).

The prognosis of TBI patients depends on various factors, including the severity of the injury, age, and sex. The epidemiology of TBI reveals significant differences between men and women. In general, men have a higher incidence of TBI compared to women, with an approximate ratio of 2:1 in many countries (7). A study by Bruns and Hauser (16) indicated that 59% of TBI cases in the U.S. occurred in men compared to 41% in women. This difference is attributed to greater male exposure to risky work situations, contact sports, and impulsive or dangerous behaviors. However, in adults over 65, the gender gap decreases due to the higher prevalence of falls in elderly women with osteoporosis and frailty (16, 17).

Over the years, the epidemiological patterns of TBI have changed. Improvements in road conditions and vehicular safety measures have reduced high-speed traffic accidents. However, the aging population has increased the incidence of TBI in older adults, mainly due to falls (18, 19). Additionally, injury mechanisms vary by sex: men have higher mortality in traffic accidents and falls from great heights, while women tend to suffer falls at ground level (10).

Extracranial complications of TBI represent a clinical concern, especially in patients admitted to the Intensive Care Unit (ICU). These complications affect various systems, including cardiovascular, renal, hematological, and infectious (20, 21). Renal and metabolic complications, such as acute renal failure, electrolyte imbalances, and hematological disorders like coagulopathies and deep vein thrombosis, are common (22). Additionally, nosocomial infections, such as ventilator-associated pneumonia and bacteremia, can complicate the patient's clinical course (22).

Regarding sex differences, studies have shown that men are more predisposed to coagulopathies and thromboembolic events, while women are more prone to urogenital infections (19, 22). Although extracranial complications do not directly predict worse outcomes, their impact on patient evolution necessitates rigorous monitoring and personalized management (22, 23). Sex differences also influence clinical outcomes of TBI. Recent research suggests that men have higher mortality rates and severe injuries compared to women (18, 19). However, women may present higher rates of long-term disability and poorer functional outcomes, especially in cases of moderate and mild injuries (7, 24–26). This has been linked to hormonal and anatomical factors, such as lower neck muscle strength and greater susceptibility to acceleration and deceleration movements (27, 28).

The management of TBI has evolved with the incorporation of advanced neuromonitoring and neuroimaging techniques, allowing for better diagnosis and treatment of patients (20). Optimizing metabolic support and personalizing treatment according to sex and other prognostic factors can improve clinical outcomes (29). It is essential that future research continues to explore the relationship between biological sex and TBI outcomes. Analyzing the impact of biological sex and gender will allow for the design of more specific and effective therapeutic strategies (29).

The aim of this study is to describe, from a biological sex perspective, the clinical and evolutionary parameters of traumatic brain injury patients hospitalized in the ICU at Hospital La Paz between 2015 and 2019.

## 2 Materials and methods

### 2.1 Study design and data collection

The data are extracted from the trauma register in the intensive care unit called “RETRAUCI.” It is an initiative led by a group of Intensive Medicine professionals in Spain and sponsored by the Trauma and Neurocritical work group of the Spanish Society of Intensive Medicine and Coronary Units. It operates on an electronic database. Currently, it includes 50 registered ICUs with 124 investigators collecting data from trauma patients via a web-based system (www.retrauci.org) (30, 31).

For this study, we conducted an observational, retrospective analysis using data from Hospital La Paz in Madrid, Spain. Ethical approval for the registry was obtained from the Ethics Committee of Hospital Universitario 12 de Octubre, Madrid (Approval Number: 12/209). The study was conducted in accordance with the STROBE guidelines, the Declaration of Helsinki, and applicable biomedical research regulations.

All patients who met the following inclusion criteria were selected for the study: patients admitted to the participating ICUs from March 2015 to December 2019 who presented isolated significant Traumatic Brain Injury, defined as an abbreviated injury scale point score (AIS) ≥ 3, were included. Patients with AIS head <3 or patients with AIS ≥ 3 in any other anatomical area were not included in the study. Data on epidemiology, acute management in the pre-hospital and in-hospital stages, type and severity of injury, resource utilization, complications, and outcomes were recorded. Patients were followed up until hospital discharge.

Following the RETRAUCI protocol (30, 31), epidemiological data were collected, including age, sex, date of trauma and ICU admission, intent, mechanism and type of trauma, as well as history of treatment with antiplatelet or anticoagulant drugs and possible substance abuse. Aspects related to pre-hospital care were also documented, such as the type of initial assistance and the need to secure the airway (IOT). Various severity indices were calculated, both physiological (Revised Trauma Score [RTS]) and anatomical (New Injury Severity Score [NISS]). During the ICU stay, various complications were monitored, including rhabdomyolysis, respiratory dysfunction, multiple organ dysfunction syndrome, intracranial hypertension, renal failure, and nosocomial infections. Resource utilization was also assessed, including the need for transfusions, number of surgical interventions (urgent and non-urgent), duration of mechanical ventilation, use of neuromonitoring, and performance of tracheotomies. Finally, the clinical evolution and final destination of the patients during their ICU stay were recorded.

### 2.2 Data analysis

Quantitative variables are described using the mean and standard deviation. Categorical variables are described with frequency distributions. The normality of the distribution was analyzed using the Shapiro–Wilks test. Categorical variables are expressed through their frequency distributions and percentages. For the inferential analysis, we used the Chi-square test for independent samples when the outcomes were categorical data, and the Mann-Whitney test to assess differences between quantitative results. All analyses were performed with SPSS v.25, and a 95% confidence interval was established.

## 3 Results

A total of 950 patients were admitted to this study based on the inclusion criteria. Most of the patients were men (722), with a mean age of 45.22 and a standard deviation of ±17.45, while the mean age of women was 45.68 with a standard deviation of 18.27.

The average ICU stay was 9.82 days (SD 19.43) (9.43 ± 14.86 for men and 11.03 ± 29.59 for women, p=0.026). The average number of post-ICU hospitalization days was 10.87 ± 19.43 (9.85 ± 14.32 for men and 14.11 ± 23.81 for women, p=0.21), and the average total length of stay was 20.71 ± 28.76 (19.30 ± 23.78 for men and 25.20 ± 40.44 for women, p=0.112). We found statistically significant differences only in terms of ICU stay.

Regarding the timing of accidents, the distribution was not similar, with a higher percentage of accidents occurring during the weekend (*p* = 0.008) and during the months of March, July, and September (*p* = 0.015). This distribution regarding the timing of accidents was consistent for both genders, with no statistically significant differences observed, as shown in Table 1.

**Table 1:** Distribution of admissions by day of the week and month of the year.

**Day of trauma**	**Men**	**%**	**Women**	**%**	**Total**	**%**
Monday	89	12.30	29	12.80	118	12.40
Tuesday	88	12.20	35	15.40	123	13.00
Wednesday	84	11.60	31	13.70	115	12.10
Thursday	91	12.60	33	14.50	124	13.10
Friday	119	16.50	40	17.60	159	16.80
Saturday	126	17.50	27	11.90	153	16.10
Sunday	125	17.30	32	14.10	157	16.50
**Month of trauma**	**Men**	**%**	**Women**	**%**	**Total**	**%**
January	57	6.01	21	2.21	79	8.31
February	53	5.59	19	2.00	72	7.58
March	89	9.38	20	2.11	109	11.48
April	53	5.59	19	2.00	72	7.58
May	57	6.01	20	2.12	77	8.10
June	52	5.48	21	2.21	73	7.68
July	73	7.70	23	2.42	96	10.10
August	59	6.22	15	1.58	74	7.79
September	74	7.80	18	1.90	92	9.68
October	57	6.01	12	1.27	69	7.26
November	49	5.17	22	2.32	71	7.47
December	49	5.17	17	1.80	66	6.95

*p*-value for day of trauma: 0.319 p-value for month of trauma: 0.619.

A statistically significant relationship was identified between gender and the mechanism of trauma production (*p* < 0.001). Most traumas occurred in men; traffic accidents and falls were the main mechanisms of unintentional trauma, while assault and self-harm were the main mechanisms of intentional trauma. The highest percentage of men were involved in vehicle or bicycle accidents, followed by falls, blows, and stab wounds. Most trauma cases resulted in contusions in men, with 649 cases compared to 216 in women, while penetrating traumas were reported in only 73 men vs. 11 women. This relationship between the type of trauma and gender was also statistically significant (*p* = 0.0149). Finally, traumas were grouped based on intentionality, considering assault and self-harm as intentional, and in this case, we found no relationship between the intentionality of the trauma and gender (*p* = 0.108). These results are presented in more detail in Table 2.

**Table 2:** Mechanisms of production and type of trauma.

**Mechanism**	**Men**	**%**	**Women**	**%**	**Total**	**%**
Motorcycle	171	23.82	23	10.17	194	20.55
Pedestrian	65	9.05	53	23.45	118	12.50
Bicycle	48	6.68	6	2.65	54	5.72
Fall	94	13.09	48	21.24	142	15.04
Accidental fall	125	17.41	47	20.79	172	18.22
Other vehicles	15	2.09	2	0.88	17	1.80
Stabbing	44	6.13	3	1.33	47	4.98
Car	76	10.58	35	15.49	111	11.76
Firearm	7	0.97	1	0.44	8	0.85
Diving accident	13	1.81	1	0.44	14	1.48
Unknown	12	1.67	3	1.33	15	1.59
Hit by object	9	1.25	2	0.88	11	1.16
Crushing	23	3.20	1	0.44	24	2.54
Bull horn	3	0.42	0	0.00	3	0.32
Other	6	0.84	0	0.00	6	0.64
Explosion	7	0.97	1	0.44	8	0.85
**Type of trauma**						
Blunt	649	89.89	216	95.15	865	91.15
Penetrating	73	10.11	11	4.85	84	8.85
**Intentionality of trauma**						
Intentional	104	14.4	44	19.4	148	15.6
Unintentional	604	83.5	181	79.7	785	82.6
Unknown	15	2.1	2	0.9	17	1.8

When analyzing the relationship between trauma and alcohol consumption, it is noteworthy that most trauma cases are not related to alcohol [76.84% in men, 90.71% in women, with a statistically significant relationship (*p* < 0.0001)]. Regarding drug abuse among people who have suffered TBI, higher consumption has also been identified in men with statistically significant differences (*p* = 0.007). As for the consumption of psychotropic drugs in this context, we see that it is present only in a small percentage, and that chronic use of these medications is higher in women than in men, although without statistically significant differences based on sex (*p* = 0.165). About 9.7% of the total patients were on some type of anticoagulant treatment prior to the TBI; the differences between sexes regarding the use of anticoagulants/antiplatelets were not statistically significant (*p* = 0.529). Detailed information on the use of alcohol, psychoactive drugs, and antiplatelet/anticoagulant treatments is shown in Table 3.

**Table 3:** Previous substance consumption.

**Substance consumption**	**Men**	**%**	**Women**	**%**	**Total**	**%**
**Alcohol**						
No	553	76.806	205	90.708	758	80.127
Yes	167	23.194	21	9.292	188	19.873
**Drug abuse**						
No	637	88.595	215	94.714	852	90.063
Yes	82	11.405	12	5.286	94	9.937
**Previous antiplatelet/anticoagulation**						
1: Antiplatelet	37	5.2	15	6.6	52	5.5
2: Anticoagulated	14	2.0	9	4.0	23	2.4
3: New antiplatelets	2	0.3	0	0.0	2	0.2
4: New anticoagulants	2	0.3	1	0.4	3	0.3
5: Dual antiplatelet	2	0.3	0	0.0	2	0.2
6: Antiplatelet + anticoagulated	7	1.0	2	0.9	9	1.0
No	650	91.0	199	88.1	849	90.3

Regarding origin, the highest percentage of patients are admitted to the unit directly through pre-hospital care (87.35%), with 92.80% using mobile ICU services. Despite this, 66.41% did not require endotracheal intubation. We found statistically significant differences based on gender regarding the type of pre-hospital care (*p* = 0.005). About 50.43% presented with instability or shock at varying levels. Table 4 presents information on the origin and characteristics of patients upon arrival at the hospital.

**Table 4:** Origin and characteristics upon admission.

**Origin and characteristics**	**Men**	**%**	**Women**	**%**	**Total**	**%**
**Origin**						
Pre-hospital	609	87.752	185	86.047	794	87.349
Emergency Room	32	4.611	14	6.512	46	5.061
Operating Room	12	1.729	2	0.930	14	1.540
Other	41	5.908	14	6.512	55	6.051
**Pre-hospital care**						
Mobile ICU	656	92.655	208	93.274	864	92.803
Non-medicalized	10	1.412	10	4.484	20	2.148
Helicopter	16	2.260	0	0.000	16	1.719
None	8	1.130	3	1.345	11	1.182
Unknown	18	2.542	2	0.897	20	2.148
**Pre-hospital IOT**						
No	468	66.477	145	66.210	613	66.414
Yes	228	32.386	72	32.877	300	32.503
Alternative Airway	8	1.136	2	0.913	10	1.083
**Hemodynamic situation**						
Stable	346	49.2	112	50.7	458	49.6
Unstable, responds to volume	162	23.0	45	20.4	207	22.4
Shock	153	21.8	50	22.6	203	22.0
Refractory Shock	42	6.0	14	6.3	56	6.1

*p* = 0.1653.

The percentage of patients with a New Injury Severity Score (NISS) >15 was 68.07% (66.76% in men and 72.25% in women). Regarding the Revised Trauma Score (RTS) <6, it was 27.50% (28.53% in men and 24.23% in women). The average NISS score was 23.12 ± 14.66 (22.88 ± 14.58 in men and 23.88 ± 14.90 in women) and the RTS was 10.55 ± 2.50 (10.53 ± 2.51 in men and 10.61 ± 2.44 in women); no statistically significant differences were found in these mean scores based on gender, analyzed using the non-parametric Mann Whitney U test (p=0.303 for the NISS score and p=0.550 for the RTS).

Regarding resource consumption, it is noteworthy that 459 people (48.37%) required some type of urgent surgical intervention (performed within the first 24 h), with trauma surgeries being the most frequently performed (21.5%), followed by neurosurgery interventions (14.5%). In the case of women, 104 urgent surgeries (45.8%) were performed, and in men, 355 surgeries (40.2%). No statistically significant differences were found regarding the performance of surgeries between genders (*p* = 0.210).

Regarding the consumption of blood products, 26.3% of subjects (25.1% of men and 30.4% of women) received at least one unit of red blood cells, and 13% (10.8% men and 20% women) received FFP. No statistically significant differences were found in the consumption of blood products based on gender (*p* = 0.280 for red blood cell transfusion and *p* = 0.063 for the use of fresh frozen plasma).

Regarding the need for renal replacement therapy, only 18 people (2.4%, 15 men and 3 women) required continuous renal replacement therapy, and 96 people (12.5%, 73 men and 23 women) required intermittent renal replacement therapy. No statistically significant differences were found in the use of this therapy between men and women (*p* = 0.937 for intermittent therapy and *p* = 0.449 for continuous therapy).

A total of 113 people required a tracheostomy (14.8%, 20 women and 93 men). In most cases (87%), a percutaneous tracheostomy was performed. No statistically significant differences were found in the need for tracheostomy between men and women (*p* = 0.217).

The average number of days of mechanical ventilation was 4.51 ± 10.96 days (4.63 ± 11.1 in men and 4.12 ± 10.4 in women). No statistically significant differences were found in the days on mechanical ventilation (*p* = 0.963) between men and women.

No significant differences were found in ICU complications, with similar percentages appearing in both genders in the requirement for decompressive craniectomies, occurrence of rhabdomyolysis, nosocomial infection, etc., as shown in Table 5.

**Table 5:** Presence of complications.

**Complication**	**Men**	**%**	**Women**	**%**	**Total**	**%**
**Decompressive craniectomy**						
No	529	92.8	165	91.7	694	92.5
Yes	41	7.2	15	8.3	56	7.5
**Rhabdomyolysis**						
No	573	81.4	180	81.4	753	81.4
Yes	131	18.6	41	18.6	172	18.6
**Brain herniation**						
No	548	77.8	177	80.1	725	78.4
Yes, requires first-level measures	69	9.8	16	7.2	85	9.2
Yes, requires second-level measures	60	8.5	20	9.0	80	8.6
Managed as donor	27	3.8	8	3.6	35	3.8
**Respiratory dysfunction (PaFi)**						
No	519	73.7	176	79.6	695	75.1
>300	98	13.9	25	11.3	123	13.3
201–300	66	9.4	16	7.2	82	8.9
101–200	19	2.7	4	1.8	23	2.5
<100	2	0.3	0	0.0	2	0.2
**Multiple organ dysfunction syndrome**						
No	638	90.6	200	90.5	838	90.6
Early	58	8.2	17	7.7	75	8.1
Late	8	1.1	4	1.8	12	1.3
**Massive hemorrhage**						
No	658	93.5	205	92.8	863	93.3
Yes	46	6.5	16	7.2	62	6.7
**Nosocomial infection**						
No	589	83.7	187	85.4	776	84.1
Yes	115	16.3	32	14.6	147	15.9

*p*-values: craniectomy p = 0.612, Rhabdomyolysis *p* = 0.985, Brain Herniation *p* = 0.709, Respiratory Dysfunction *p* = 0.452, MODS *p* = 0.722, Massive Hemorrhage *p* = 0.714, Nosocomial Infection χ^2^=0.371 *p* = 0.543.

Table 6 shows the use of neuromonitoring (monitoring of ICP, jugular saturation, NIRS, and PtiO2), indicated only in individuals with a Glasgow score ≤ 8 (25.7% of the study population: 242 people, 192 men and 50 women). No statistically significant differences were found in the use of neuromonitoring systems between men and women. The average number of days of ICP use was 1.04 ± 3.54 (1.17 ± 3.88 in men and 0.62 ± 2.02 in women), with no statistically significant differences found (p=0.214).

**Table 6:** Use of neuromonitoring.

**Neuromonitoring type**	**Men**	**%**	**Women**	**%**	**Total**	**%**
**ICP monitoring**						
Intraparenchymal	49	25.5	13	26	62	25.6
Intraventricular	22	11.4	3	6	25	10.3
Both	7	3.6	3	6	10	4.1
NIRS	3	1.6	1	2	4	1.7
PtiO2	32	16.7	5	10	37	15.3

*p*-values: ICP Monitoring *p* = 0.544, NIRS *p* = 0.246, PtiO2 *p* = 0.581.

Table 7 presents the evolution of the patients. No significant differences were found between genders regarding their evolution in the ICU, with the majority in both cases being discharged to the ward as the patients' destination. Mortality was higher in women (14.2%), with the main causes of death being brain herniation and MODS, also without statistically significant differences between sexes. We found statistically significant differences only in terms of hospital evolution.

**Table 7:** Patient evolution by gender.

**Evolution in ICU**	**Men**	**%**	**Women**	**%**	**Total**	**%**
Discharge to ward	627	87.6	194	85.8	821	87.1
Transfer to another ICU	14	2.0	5	2.2	19	2.0
Death	50	7.0	20	8.8	70	7.4
Death and donation	25	3.5	7	3.1	32	3.4
*p = 0.325*						
**Hospital evolution**						
Discharge to Rehab Hospital	67	9.4	19	8.4	86	9.1
Discharge to reference center	27	3.8	18	8.0	45	4.8
Death	78	10.9	32	14.2	110	11.7
ICU readmission	4	0.6	3	1.3	7	0.7
Home discharge	540	75.4	154	68.1	694	73.7
*p = 0.031*						
**Therapeutic effort limitation**						
No	674	94.4	208	92.4	882	93.9
Yes	40	5.6	17	7.6	57	6.1
*p = 0.285*						
**Cause of death**						
Exsanguination	2	2.7	0	0.0	2	2.0
Brain herniation	40	53.3	13	48.1	53	52.0
Multiorgan dysfunction syndrome	15	20.0	9	33.3	24	23.5
Others	18	24.0	5	18.5	23	22.5
*p* = 0.460						

## 4 Discussion

Traumatic brain injury (TBI) is a major cause of hospitalization and mortality worldwide, which can lead to significant extracranial complications, particularly in ICU patients, where concurrent injuries in other organ systems complicate clinical outcomes (20, 21, 32, 33). In this study, the epidemiological and clinical parameters of TBI have been analyzed based on biological sex, aiming to identify differential patterns in presentation, management, and clinical evolution of patients (20, 21, 32, 33).

Sex differences in patient characteristics upon admission, care received, and outcomes have been extensively studied in cardiology and critical care, but less so in neurosurgery, mainly focusing on mortality. At the same time, postoperative complications have been little studied (34). In studies developed in critical care settings, there is talk of the possibility of receiving adequate treatment due to gender biases (35, 36). Differences persist in the lack of gender representation in TBI research (37), as occurs in other pathologies regarding survival by sex in acute pathologies (38–40) or in the presence of risk factors (41). Moreover, the low representation of women in previous studies—a phenomenon known as the “Yentl Syndrome”—could impact the external validity of the obtained results (42–45), and at the same time, the sample size of the studies should be considered, as Gupte et al. indicates, in studies with small samples, worse results are reported in women, while studies with large samples tend to find that women have better recovery in intensive care settings (27).

Our results show that 76% of patients admitted to the ICU for TBI were men, in line with previous studies (46); however, average stays are longer in women, both in the ICU and in total hospitalization time, although statistically significant differences were only found in the average ICU stay. Various studies have indicated a higher likelihood of ICU admission for men, as well as more severe injuries in this population (47–49). Age and sex disparities are critical factors for understanding TBI outcomes, as research indicates that men generally present higher injury rates and worse outcomes compared to women (18, 22). Nonetheless, some research suggests that, despite this trend, women may present greater long-term sequelae (2, 50, 51). Additionally, studies like Breeding (52) have identified higher hospital mortality in men, possibly influenced by sociocultural factors and differential biological responses to trauma (53). Epidemiological studies show that men have a higher prevalence of TBI compared to women, with an approximate ratio of 2:1 in many countries (7). In a study conducted by Bruns and Hauser (16), it was observed that 59% of TBI cases in the United States occurred in men, compared to 41% in women. This pattern has been replicated in different regions of the world and has been attributed to factors such as greater exposure of men to risky work situations and impulsive or dangerous behaviors (6, 7).

This male predominance is mainly observed in young and adult ages, especially in the age group of 15–24 years, a period characterized by greater participation in risky activities such as sports, motor vehicle driving, and interpersonal violence, although this gap has decreased in recent years (54). On the other hand, the progressive equalization of women in different professional and recreational fields with a higher probability of suffering TBI should be considered. For this reason, various authors increasingly consider that biological sex should be taken into account as an essential variable in the context of care outcomes (25, 26, 55); however, there are still few publications that describe the consequences of TBI differentiated by sex (56). Nonetheless, in people over 65 years old, the gender gap in TBI incidence tends to close, partly due to the higher prevalence of falls among older women, especially those with osteoporosis and frailty issues (16, 17).

Regarding the distribution of admissions, our study found an increase in the frequency of TBI occurrence on weekends and in the spring and summer months, consistent with previous studies (57, 58). On the other hand, most patients admitted to our hospital come from pre-hospital care and have been treated in out-of-hospital critical care units. The early transfer of patients with traumatic brain injury (TBI) to specialized reference units is a fundamental strategy to improve survival and functional outcomes. Various clinical practice guidelines recommend that patients with moderate or severe TBI be transferred directly to centers with neurosurgical capacity and specialized intensive care, minimizing the time until definitive intervention (59). Evidence supports this recommendation: observational studies have shown that TBI patients who are directly transferred to level I trauma centers have lower adjusted mortality compared to those who first receive care in non-specialized hospitals and are subsequently transferred (60, 61).

Furthermore, secondary transfers can lead to delays in critical interventions such as surgical decompression or intracranial hypertension control, increasing the risk of irreversible neurological deterioration (61). Therefore, establishing pre-hospital protocols that prioritize direct transfer to reference units, according to well-defined clinical criteria, is key to more effective, equitable, and evidence-based care (59, 62). The early transfer of patients with traumatic brain injury (TBI) to specialized centers is essential to improve clinical outcomes. Therefore, implementing protocols that prioritize direct transfer to reference units is fundamental to optimizing care and outcomes in TBI patients (59, 60).

One aspect highlighted in this study is the differential distribution of trauma mechanisms according to biological sex. While men are primarily involved in traffic accidents and falls, women present a higher proportion of intentional traumas related to self-harm. This point coincides with previous studies that have identified that women are more likely to suffer injuries as passengers in traffic accidents or as a consequence of suicide attempts (47–49). Other studies like Teterina's indicate that men have more diagnoses associated with high-risk activities and occupational accidents, and women have more diagnoses related to partner violence and physical abuse (29). Findings from the Japan Trauma Data Bank highlighted that men generally experience more severe injuries compared to women, which may be attributed to a higher prevalence of high-impact traumas and alcohol intoxication among male patients. In that study, it was found that, while falls are the leading cause of TBI among older adults, older women were more frequently affected by falls from ground level, while falls from greater heights were more common in older men (18). The epidemiology of TBI has shown changing patterns over the years. For example, while high-speed traffic accidents have decreased due to better road conditions and vehicle safety features, population aging presents new challenges related to TBI risk (18). Current data indicate that older adults are increasingly vulnerable to falls, which are a significant factor in TBI incidence in this demographic group.

Additionally, injury mechanisms differ by sex, and specific activities, such as being a backseat passenger during an accident or suffering falls from significant heights, correlate with higher mortality rates among men (10). Given the significant percentage of injuries from traffic accidents, the population should be made aware of the use of safety measures and precautions to minimize the impact of traumatic brain injuries (63). As new epidemiological patterns emerge, such as the increase in accidents related to electric vehicles, it is crucial to consider their inclusion in future studies (46), as well as consider the inclusion of others, such as TBIs due to gender-based violence, which due to their added physical and psychological complications in the long term, may be an aspect to study (63, 64). Previous studies have indicated that there are epidemiological and clinical differences according to biological sex, justifying the need for comparative studies that analyze the impact of TBI from this perspective (32, 33).

Regarding substance use in the context of TBI, a higher prevalence of alcohol and drug use has been identified in men (23.19% and 11.40%, respectively) compared to women (9.29% and 5.2%, respectively). Previous research has pointed out that alcohol consumption before trauma can negatively influence evolution and the severity of sequelae (65–67). Additionally, the literature suggests that substance use may increase during the recovery period, affecting long-term quality of life (23, 67). We also found a higher proportion of women with chronic psychotropic use compared to men (9.25% vs. 5.68%). Studies like Jakob's indicate that stimulant use may be related to prolonged hospital stays (68), while other works have described an increase in psychotropic use after TBI recovery, linked to symptoms of post-traumatic stress (69, 70).

Regarding transfusions, their use has been similar in both sexes, finding low rates, although in some cases multiple units of red blood cells or FFP were needed, probably in people with polytrauma at another level. It should be noted that recommendations on blood transfusion in TBI patients focus on maintaining optimal hemoglobin levels to ensure adequate cerebral oxygenation without incurring risks associated with unnecessary transfusions (71). Studies indicate that maintaining hemoglobin between 7 and 9 g/dL is generally safe and effective in TBI patients, avoiding complications that may arise from excessive transfusions. However, it is essential to individualize the decision to transfuse, considering factors such as the patient's hemodynamic stability, the presence of concomitant injuries, and signs of tissue hypoxia. Additionally, caution should be exercised with coagulopathies associated with TBI, as they may be exacerbated by inadequate transfusions (71). Nonetheless, the study by Taccone that evaluates liberal transfusion strategies vs. restrictive strategies finds a lower probability of having an unfavorable neurological outcome in patients with liberal strategies (72). Continuous monitoring and detailed clinical evaluation are fundamental to determining the need and appropriate timing for transfusion in these patients.

Regarding the need for pre-hospital orotracheal intubation, no significant differences were observed between sexes, as one-third of patients requiring this intervention in both groups. The lack of standardization in intubation protocols could influence these results, given that some studies recommend early intubation in situations of hypoxia and airway obstruction (32, 33, 73–75), indicating that early pre-hospital intubation may reduce morbidity and mortality in these patients.

Understanding the complex interaction between sex, injury severity, and extracranial complications is fundamental, as recent evidence suggests that although these complications do not independently predict adverse outcomes, they can exacerbate pre-existing intracranial injuries, thereby complicating the recovery process (22). This underscores the importance of early detection and timely intervention in managing these complications, with the potential to significantly improve patient prognosis (27). The use of severity assessment scales such as NISS or RTS allows for initial care adjustment (76, 77) and a more personalized approach to TBI patient care, complemented by comprehensive monitoring strategies. In this context, continuous neuromonitoring and the use of advanced neuroimaging techniques, such as computed tomography (CT), are increasingly playing a relevant role in the diagnosis and prognosis of TBI patients, facilitating early interventions and improving overall clinical outcomes (20). In our case, the percentage of patients with a Glasgow score below 8 was 25.7%, and neuromonitoring results were similar to previous studies, where injury severity and the need for urgent neurosurgery increased the possibility of neuromonitoring (78, 79).

Extracranial complications in traumatic brain injury (TBI) patients admitted to the ICU are frequent and affect multiple systems, significantly contributing to morbidity and mortality (19, 22, 80, 81). In our study, no statistically significant differences were found in the occurrence of complications between sexes. Among the complications in the literature, coagulopathies, hemorrhages, thromboembolisms, and infections, such as ventilation-associated pneumonia, as well as iatrogenic complications, are included (19, 22). Although factors such as age, frailty, and Glasgow Coma Scale (GCS) score influence outcomes more than sex, differences between men and women remain relevant. Men present a greater predisposition to coagulopathies, while women show a higher incidence of urogenital infections, especially in cases of isolated TBI (22, 27). Some studies indicate that women have worse outcomes after mild or moderate TBI, although they may recover better in severe cases due to hormonal and immunological factors {27, 29, 54, 79}. Additionally, older men present more neurological and systemic complications, with higher mortality in old age (27, 29). These differences reinforce the need for sex-sensitive clinical care and future research that clearly distinguishes between the impact of biological sex and gender to optimize treatments (29).

This study, even though providing a description and analysis of a significant number of patients with Traumatic Brain Injury, offering an objective view of the characteristics of patients admitted for this condition in a top-tier hospital, has several limitations, such as being conducted using data from a single hospital center.

Moreover, we must consider that this is a cross-sectional study, where confounding factors that could influence clinical outcomes (comorbidities, polytrauma, etc.) may arise.

It is also important to note that the objectives of the RETRAUCI study did not include examining gender-based differences among participants, so variables that could better represent these differential characteristics were not included. Nevertheless, the results provide us with a fantastic overall scientific perspective.

Finally, we present data collected five years ago, acknowledging the delay caused by the COVID pandemic, which prevented these findings from being published earlier. However, the results contribute significantly to scientific knowledge. Future RETRAUCI studies should be conducted to determine if the profile has changed in recent years.

## 5 Conclusion

Our findings reveal that biological sex plays a critical role in the clinical trajectory of ICU patients with traumatic brain injury. While men are more frequently exposed to high-risk mechanisms such as traffic accidents, women experience longer ICU stays and higher psychotropic drug use, suggesting distinct clinical and social challenges for each group. Despite similar rates of complications and interventions, these differences imply that uniform care pathways may inadequately address patient-specific needs.

These insights challenge the current one-size-fits-all approach in TBI management and call for more nuanced, sex-informed clinical practices. Integrating biological sex as a core variable in care protocols and research design is not merely an academic detail—it is essential for ensuring both equity and efficacy in treatment. To fully understand the long-term consequences of these sex-based differences and to improve clinical outcomes, further prospective and sex-stratified research is required.

Biological sex significantly shapes the clinical course of ICU patients with traumatic brain injury. Men are more often injured by high-risk mechanisms, while women face longer ICU stays and greater psychotropic drug use, reflecting distinct clinical and social challenges.

These findings highlight the limitations of uniform care pathways and underscore the need for sex-informed practices.

Incorporating sex as a core variable in protocols and research is essential to improve equity, efficacy, and long-term outcomes.

## Data Availability

Raw data are available from the corresponding author upon reasonable request.
